# Remote PEERS^®^ for preschoolers: A pilot parent-mediated social skills intervention for young children with social challenges over telehealth

**DOI:** 10.3389/fpsyt.2022.1008485

**Published:** 2022-11-29

**Authors:** Reina S. Factor, Leila Glass, Daliah Baertschi, Elizabeth A. Laugeson

**Affiliations:** Department of Psychiatry and Biobehavioral Sciences, Semel Institute for Neuroscience and Human Behavior, University of California, Los Angeles, Los Angeles, CA, United States

**Keywords:** autism, preschool, social skills, telehealth intervention, PEERS^®^

## Abstract

**Introduction:**

Social differences characteristic of autism spectrum disorder (ASD) and other developmental disabilities are evident in early childhood and are associated with later difficulties. Unfortunately, there is a paucity of evidence-based interventions explicitly targeting social skills development for young children, few actively integrate parents and caregivers, and even fewer have remote models. The importance of providing accessible, tailored services for families in the wake of the COVID-19 pandemic, prompted the creation of a parent-mediated telehealth version of Program for the Education and Enrichment of Relational Skills (PEERS^®^) for Preschoolers (P4P), a pre-existing, evidence-based social skills intervention for children 4–6 years focused on making and keeping friends.

**Method:**

This methodological paper documents the implementation, feasibility, and satisfaction of a novel telehealth group-based delivery of P4P.

**Results:**

Qualitative results indicate acceptable feasibility and satisfaction. Additionally, following completion there was an increase in parental confidence in social coaching and increased use of child social skills.

**Discussion:**

Future work will evaluate quantitative outcomes and comparisons between delivery methods (e.g., telehealth vs. in-person).

## Introduction

Social difficulties are common across a number of diagnostic groups, including attention-deficit/hyperactivity disorder (ADHD), autism spectrum disorders (ASD), and mood disorders (e.g., anxiety, depression), often worsening throughout development (1, 2). Social differences are often evident in early development in autistic^1^ individuals (4) and those with other developmental concerns (5), and can result in longstanding negative outcomes. As the theory of the double empathy problem explains, these social-communication differences create barriers both for autistic individuals and for their non-autistic peers, which may result in autistic children having fewer friends, friendships of diminished reciprocity, and higher loneliness than their non-autistic peers, though it is unclear if the same findings are true for neurodivergent dyads (6–9). Many autistic individuals, and their families, seek effective social skills interventions to help improve their social communication abilities and learn the skills to make and keep friends.

To meet this patient and family driven clinical need, evidence-based social skills groups focused on making and keeping friends have been developed for adolescents and young adults with difficulties making and keeping friends (10), including the Program for the Education and Enrichment of Relational Skills (PEERS^®^) (11–13). Numerous studies have shown an increase in social functioning (13), with gains maintained 1–5 years post-PEERS^®^ intervention (14). Despite the success of social skills programs like PEERS^®^, research suggests that few interventions explicitly target social skills in young children as a primary intervention focus even though social challenges are often apparent to caregivers, teachers, and peers by 4–6 years of age (15–17). Without effective intervention, social difficulties can increase risk for aggression, peer rejection, social dissatisfaction, and academic failure, among other problems in children (18). Therefore, it is essential to intervene at a young age. Building on the positive results of the PEERS^®^ group-based social skills programs for other age groups, PEERS^®^ for Preschoolers (P4P) was originally developed as an in-person, parent-assisted intervention for younger children (ages 4–6 years). Since its inception, P4P has demonstrated initial positive outcomes (e.g., increased social skills, reduction in problem behaviors) (16, 19–21).

While initial P4P research findings are promising, access to evidence-based services remained elusive for many families who could not travel to locations where it was offered for various reasons or could not attend due to childcare issues or other systematic barriers. Additionally, access to in-person services further diminished for families of young children, as a result of COVID-19, when treatments largely moved to online delivery. Although the adolescent and young adult versions of PEERS^®^ seamlessly moved to remote delivery, programs like P4P, which target young children, collectively ground to a halt in early 2020. Families were left with minimal access to effective intervention, despite being a group with significant need and the greatest potential for benefit based on the support for early intervention (22). In consideration of providing services to this population, it is acknowledged that capturing the attention and motivation of young children over video-conferencing platforms is a significant challenge and it is particularly difficult for young children to learn social skills and participate in a behavioral intervention using a remote modality. Yet, the importance of providing effective social skills intervention for families in need, especially during a global pandemic, was of utmost importance. This challenge and mandate prompted the creation of a remote option for P4P. Consideration of best methodology led to the development of a modified P4P intervention utilizing a parent-mediated (i.e., parent-only) social skills telehealth group-based model. This pilot feasibility paper aims to outline the methodological implementation of P4P, and to investigate acceptability, feasibility, and initial satisfaction of the novel telehealth parent-mediated version of P4P with the intention of improving social skills for young children with difficulty making and keeping friends.

### Need for social skills interventions for young children

Despite the importance of early interventions (23), few interventions explicitly address the development of social skills in young autistic children, or those with social skill difficulties, as a *primary* target of change (15, 24). While interventions may include elements of social skills, these treatments are often conducted in one-on-one settings with an emphasis on social communication (25) rather than developing ecologically valid social skills as a *primary* outcome (26, 27). Previous research has indicated difficulty in identifying these skills to target within the social skills intervention context (28). This work suggests that it is difficult to identify and treat ecologically valid social skills, especially at this age, for a variety of reasons [e.g., variability in development (language/cognitive), variability in contexts, cultural factors] (29). Thus, there is a need for improved access to evidence-based, ecologically valid social skills interventions for young children to enhance these skills and begin teaching important skills from a young age.

### Importance of caregiver mediated intervention for young children

Not surprisingly, for children under age 6, interventions targeting different areas of functioning are often parent-mediated or include parental involvement (30). Including parents in treatment can improve generalization of skills to other settings, enhance outcomes, and increase the maintenance of gains (15). Different treatment models for integrating the caregiver exist for autism-specific interventions. Manualized caregiver-administered or implemented interventions can be complex and require extensive training, expertise, and high fidelity to be effective (31). Caregiver-mediated interventions teach caregivers how to employ strategies for supporting specific behaviors, such as promoting social engagement. This method of intervention has been found to alter behaviors that parents, teachers, other caregivers, and the child may find distressing (32). Unlike psychoeducation or caregiver-integration intervention models, which may outline core information without providing specific strategies or hands on training, caregiver-mediated or implemented interventions teach caregivers to deliver treatment techniques with the child, and support caregivers’ practice with the child actively in session and out of session (33).

While parent-mediated treatments are increasing in the literature, there is still a dearth of social skills interventions that actively integrate parents and caregivers (34), despite the important role they play in social development, especially at a young age (28, 35, 36). Therefore, a program designed for young autistic children and those with social difficulties, such as P4P, that specifically targets social skills and includes caregiver-assistance is critical.

### The program for the education and enrichment of relational skills^®^ for preschoolers program

To provide a bit more background, the PEERS^®^ program is one of the only evidence-based, parent-assisted social skills interventions for autistic adolescents and young adults (12, 13, 37). The program employs empirically supported methods of social skills instruction (i.e., didactic instruction, role-play demonstrations, behavioral rehearsal, generalization outside of session) that can be used transdiagnostically (e.g., ASD, ADHD, mood disorders, adjustment disorder). In addition to the patient group, PEERS^®^ also includes a simultaneous social coaching group that includes psychoeducation, review of social skills, and social coaching strategies. PEERS^®^ has been found efficacious across multiple randomized controlled trails (RCTs) for adolescents and adults (13, 38–40).

The PEERS^®^ program was adapted for younger children in need of social skills intervention (19–21). The original P4P program addresses similar tenets using analogous methods of instruction as other PEERS^®^ programs, but in a developmentally appropriate manner (e.g., use of puppet shows for the child didactic lesson, homework adjusted to appropriate skills) with in-person treatment delivery for children and their parents. In addition to a concurrent but separate parent training component, P4P includes parent-coached play at the end of each session to facilitate skill acquisition using *in vivo* performance feedback for both parents and children. During these parent-coached play activities, parents are given opportunities to coach their children employing the targeted social skills, while receiving coaching from the treatment team during mock playdates with other group members. To promote generalization, parents are given weekly homework assignments to create opportunities to practice skill usage with their children, including playdates with children unaffiliated with P4P. In addition to enhancing skill development and generalization across settings, these assignments facilitate frequent and authentic interactions and engagement, as well as reciprocity between parents and children. An initial RCT indicated improvements in social skills and a reduction in problem behaviors (21) and maintenance of treatment gains 1–5 years post-P4P (16, 41). Similar gains have been revealed in other studies (20) and also in examining other PEERS^®^ programs (14).

The autistic community and autism allies (i.e., parents and caregivers of young children) were involved in the development of the intervention during previous stakeholder focus groups with program developers through ongoing feedback regarding feasibility of the group meetings (e.g., consistency and timing of meetings, locations). Further, the PEERS^®^ developers have a long history of involving autistic self-advocates and stakeholders in the development and testing of all PEERS^®^ social skills interventions, including P4P.

### Telehealth program for the education and enrichment of relational skills^®^ for preschoolers program

The need for novel methods for social and emotional interventions became especially salient during the COVID-19 pandemic (42), building on already established need for a variety of systemic and access issues. Over the past decade, there have been several reviews examining technology and computer-assisted interventions to teach social skills (43, 44). These studies reveal mostly positive results (45, 46). However, the majority of these interventions were designed for adolescents and young adults, with few accessible to young children.

In considering advantages of these technological platforms for service delivery, they do allow for a more far-reaching access to intervention, with the ability for those in disparate or more remote geographic areas and those who experience service disparities, perhaps related to race or socioeconomic status, to indeed take advantage of such services (47). Such disparities are prominent in autism-related services, with fewer service providers specializing in ASD available in rural and low-income areas (48). Research has indicated a number of these telehealth services for autism included parent-mediated interventions and applied behavior analysis (ABA) approaches for young children, as well as some social skills programs using video technology (49–51). Further, these studies suggest positive outcomes for ABA interventions, Cognitive Behavioral Therapy (CBT) interventions, and targeting problem behavior (52, 53). Additional research has indicated that digital technologies may empower families by providing needed services that otherwise might be limited and also enhance social skills interventions (54).

In the midst of the move toward more telehealth-based service delivery methods, the COVID-19 pandemic has challenged mental health practitioners to provide patient care in novel formats (35). While previous research suggests telehealth delivery of the PEERS^®^ social skills intervention for autistic adolescents results in equivalent treatment outcomes to in-person groups (47, 55), providing P4P instruction over telehealth directly to young children was not considered clinically viable and therefore, not explored. However, social skills coaching for parents *via* telehealth was not only considered feasible, but warranted, especially given the strong evidence-base of telehealth delivery as well as the substantiated need for this clinical service.

This pilot methodological paper aims to outline the creation and implementation of a parent-mediated telehealth version of the PEERS^®^ for Preschoolers (P4P) Program, a 16-week parent-only intervention for young children with social difficulties. The creation and adaptation of the P4P program follows the consideration of the FRAME reporting on adaptations of evidence-based interventions in guiding this work, as well as previous PEERS^®^ telehealth programs to ensure best-practices were employed (56). Specifically, we outline the methodology for adapting and implementing the P4P telehealth program, a review of qualitative parent satisfaction data to address acceptability, feasibility, and subjective response, and recommendations for future research. The creation and successful implementation of telehealth P4P will allow for greater dissemination to improve social functioning in young children using remote delivery techniques. This study serves as part of a larger ongoing P4P telehealth intervention study.

## Materials and methods

### Participants

The pilot methodological adaptation of P4P as a telehealth group included three cohorts consisting of 30 parents and 1 grandparent of children between the ages of 4 and 6 (*M*_*age*_ = 4.93; *SD* = 0.83). Each cohort had between 10 and 12 parents consistently attend. All children were reported to have difficulties making and keeping friends by their parent(s). Though the majority of participants reported social concerns before COVID-19, a few noted that the changes with school and quarantine regulations amplified social difficulties. Participants were recruited through widespread email, social media, recruitment flyers, and word of mouth. Demographic characteristics of the sample that completed the intervention can be found in Table 1.

**TABLE 1:** Descriptive statistics for categorical variables of interest.

Variable	Percentage (*n*)
**Child sex**	
Male	73.3 (22)
Female	26.7 (8)
**Diagnoses**	
Autism spectrum disorder	63.3 (19)
ADHD	10.0 (3)
Anxiety	10.0 (3)
Other	10.0 (3)
None	16.7 (5)
**Child ethnicity**	
Caucasian	46.7 (14)
African American	3.3 (1)
Asian American	20.0 (6)
Latino/Hispanic	6.7 (2)
Mixed race	16.7 (5)
Other	6.7 (2)
**Parent/caregiver Marial status**	
Unmarried	3.3 (1)
Married	86.7 (26)
Divorced	6.7 (2)
Separated	3.3 (1)
**Highest level of schooling completed by parent/caregiver**	
Associate’s degree	3.3 (1)
Bachelor’s degree	30.0 (9)
Master’s degree	40.0 (12)
Doctoral degree	26.7 (8)
**Parent/caregiver occupation**	
Unemployed	36.7 (11)
Part-time employment	23.3 (7)
Full-time employment	40.0 (12)

All families completed a phone screen with a clinical coordinator and submitted a brief video of their child to assess for phrase speech (sentence-speech of four or more words), which was evaluated by the intake clinician. If the video was deemed appropriate, parents completed a 1.5 h clinical intake with a licensed psychologist or postdoctoral fellow to assess for eligibility and provide information about the program. Inclusion and exclusion criteria were consistent with in-person P4P groups. Specifically, children were required to have adequate expressive (e.g., at least four-word phrases) and receptive language (e.g., able to understand P4P instructions related to skills taught each week) and both parents and children were fluent in English. Participants were required to have access to devices that would support video conferencing (i.e., Zoom) for telehealth sessions. Exclusion criteria included an active child medical problem (e.g., unstable seizure disorder), severe child mental health problems (e.g., psychosis), or if the child was unable to be maintained on medication over treatment. The study was approved by the university’s Institutional Review Board and input from stakeholders within the autistic community was previously provided.

### Methodological procedure for telehealth P4P

Eligibility appointments included parental consent, assessment of parent motivation to participate, identification of social skill goals and presenting concerns, and background history of the child and parent. Parent motivation was assessed through clinical intake interview utilizing clinical judgment and questions regarding interest and availability for the group. Specifically, questions were posited about whether this was the right time or the correct treatment modality as well as whether it was a treatment priority for the family in the broader context of the child’s situation. Detailed information about P4P structure was outlined and questions were answered. Parents were notified data collected could be used for publication and though sessions were not recorded, their comments would be written down for qualitative analysis. Determination was also given to whether social skills was a treatment priority or whether other goals should take precedent. No families were excluded based on these criteria.

Weekly parent-mediated P4P telehealth groups consisted of about 12 families [some had multiple caregivers/partners per family attend, (e.g., grandparent, both parents)], two-three group leaders (licensed clinical psychologists and postdoctoral fellow), and two research coordinators. All treatment team members were trained and supervised on P4P by the PEERS^®^ developer (EAL) and all were PEERS^®^ Certified Providers. A 45-min supervisory case conference occurred before each session, where clinical concerns were discussed with the team, the corresponding didactic lesson was reviewed, and homework assignments (i.e., video demonstrations of at-home behavioral rehearsals) were viewed to determine appropriate feedback. Sessions were delivered *via* HIPPA-Compliant Zoom, previously deemed secure regarding confidentiality and effective treatment administration. This platform also allowed for break-out rooms for smaller group discussions, moderated group chat, and screen-sharing to view video demonstrations and presentation of didactic materials.

#### Telehealth P4P caregiver group structure

Parents were given weekly assignments to practice newly learned skills with their children, which included the secure submission of video recordings of brief play interactions (i.e., 1–2 min) focused on the weekly skill using P4P social coaching strategies and buzzwords (i.e., key terms) from the corresponding weekly lesson. Videos were reviewed prior to case conference and discussed clinically as a team to enhance feedback for each family. The intervention comprised 16 weekly 90-min parent groups delivered over Zoom using the following format:

##### Homework review (40–60 min)

The first 5–10 min focused on a full group discussion about homework assignments, including enrollment in playgroups and the facilitation of playdates. This time together was lengthened after week 7, when playdates were assigned for homework (20–30 min). An example of homework completion for 1 week might entail attendance, practicing the skill of the week (e.g., asking a friend to play), submitting a video demonstrating the skill of the week, having a playdate, and enrolling in a playgroup. Assignments slightly changed with each new skill, and adding playgroups and playdates, though otherwise remained consistent. The next 20–30 min involved smaller group discussion of homework assignments in Zoom breakout rooms in which parents were split into two smaller groups, each with one group leader and one research coordinator, to facilitate greater discussion and feedback while viewing homework video recordings of child behavioral rehearsals of targeted social skills. Video recordings were paused throughout review to provide virtual *in vivo* feedback and social coaching tips from the treatment team to mimic in-person coaching feedback using the “bug in the ear” technique that was implemented in the original P4P in-person format. This allowed for greater individualization of feedback, as each family was able to ask specific questions and leaders could help them navigate and troubleshoot difficult situations. This also allowed for feedback regarding specific situations relevant to each family.

Additional time was allotted to provide social coaching guidance for identifying and joining playgroups, setting up playdates, and practicing skills across settings. Parent social coaching highlighted the P4P technique known as the *4 P’s*: priming (form of cognitive rehearsal to prepare a child to practice skills immediately before a social opportunity by reviewing the rules and steps), prompting (gentle reminder to use a particular skill in the moment through buzzwords and other keys terms), praising (complimenting a child when they use or attempt to use a particular skill), and providing corrective feedback in the form of a praise sandwich (start with praise using buzzwords, then give feedback using buzzwords, end with a general statement of praise). Parents were encouraged to utilize these social coaching strategies while interacting with their children.

##### Didactic lesson (20–40 min)

Upon completion of individualized homework review in smaller breakout groups, all parents rejoined the main Zoom room for the didactic lesson as a larger group. Didactic lessons were delivered by group leaders using PowerPoint presentation slides of material. Topics and content were consistent with in-person P4P groups, which covered child skills related to making and keeping friends, including (but not limited to) meeting and greeting friends, sharing and taking turns, being a good sport, and appropriate body boundaries. Child didactic lessons included concrete rules and steps of ecologically valid social skills using buzzwords and key terms. Video puppet shows (which were also to be viewed with their children at a later time) were shown to parents to highlight the use or misuse of targeted skills. Parent didactic lessons focused on social coaching skills such as how to find appropriate playgroups and setting up playdates [see Park et al. (21) for completed review of lesson content]. To support greater understanding of social coaching techniques, parents also viewed video demonstrations of parent-coached play interactions related to the weekly skill using the *4 P’s* (i.e., a video of a parent coaching their child on the corresponding skill by providing priming, prompting, praise, and corrective feedback using a praise sandwich). Parents were often asked questions to answer aloud or in the chat feature of Zoom to maintain engagement and ensure comprehension.

##### Homework assignment (10 min)

**FIGURE 1:**
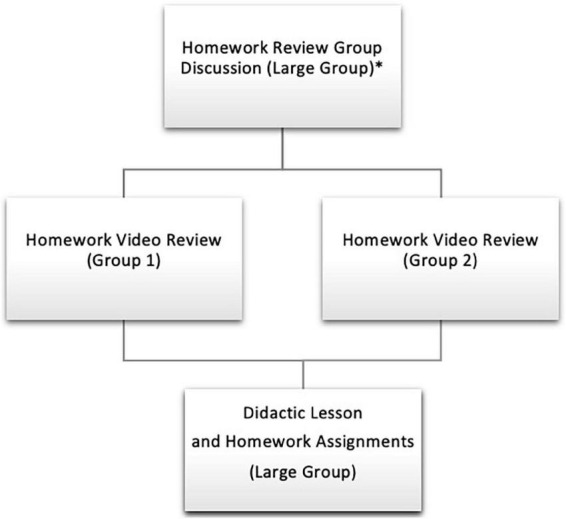
Group structure. *Following week 6 when playdates are assigned, the initial homework review group discussion is slightly longer to cover playdate homework check-ins (in addition to general concerns and updates).

Sessions concluded with a summary of assignments for the following week. Parents were given time to ask questions and troubleshoot any foreseen challenges with completing the assignments. Additionally, throughout the sessions, parents utilized the chat function to ask questions and engage in discussion both with the leaders and other parents. This led to a supportive and nurturing group dynamic, as parents were able to communicate during the group. Of note, families were not allowed to fraternize during the P4P program as to prevent any social difficulties. However, they did often exchange contact information at the conclusion of the group. See Figure 1 for a visual group structure.

##### Parent materials

PDF handouts of the slides with all content for both the child and the parent lesson, as well as video puppet shows to be viewed with children, were emailed to all parents after each session. *Parent Coached Play Cards* were provided weekly to highlight examples of how to use the *4 P’s* in relation to the targeted skill. Additionally, *Parent Homework Sheets* were also distributed for parents to monitor their completion of assignments (for their own tracking) and to note the use of the *4 P’s* through their social coaching (see Table 2).

**TABLE 2:** Program for the education and enrichment of relational skills (PEERS^®^) for preschoolers lesson content.

Week of intervention	Lesson content
Session 1	Listening to and following directions
Session 2	Meeting and greeting friends
Session 3	Sharing and giving a turn
Session 4	Asking for a turn
Session 5	Keeping cool
Session 6	Being a good sport
Session 7	Show and tell
Session 8	Don’t be bossy, be flexible
Session 9	Asking a friend to play
Session 10	Joining a game
Session 11	Asking to play something different
Session 12	Asking and giving help
Session 13	Stay in your own space
Session 14	Using an inside voice
Session 15	Review I (+ Disclosing a diagnosis to another parent)
Session 16	Review II and graduation (+ Disclosing a diagnosis to your child)

#### Parent-child practice

To ensure child learning of targeted social skills, video demonstrations of scripted didactic puppet shows (which were previously shown in-person in the child groups) were video recorded for telehealth delivery. Parents were instructed to watch these videos with their children, which not only highlight the child weekly lesson, but also include perspective taking questions to enhance social cognition (e.g., “What was that like for Kelly Cow?” “What did Kelly Cow think of Larry Lion?” “Would Kelly Cow want to play with Larry Lion again?”). Parents were told to watch these videos with their children prior to recording the video for the skill of the week. One unexpected result of the development of the video puppet shows was that parents and children were able to watch and re-watch these instructional videos to teach and reinforce skills as well as use videos as priming for recording their own video to send in for homework. Videos outlining skills exist for other PEERS^®^ groups and are available online. However, until P4P telehealth groups, these video role plays (i.e., video puppet shows) were not yet available for parents. Thus, this additional resource was provided and allowed further review of skills at home.

### Data collection

To establish acceptability and feasibility, qualitative data were collected by the trained research coordinators by taking notes during the 16-week P4P intervention through solicited and unsolicited parent feedback provided during the sessions and more formally through an anonymous Parent Satisfaction Questionnaire given at the end of the program (see Supplementary material). Results of the qualitative feedback presented in this paper were quotes from the Satisfaction Questionnaire and throughout the sessions. Homework completion, participant attendance, and attrition rates were collected weekly by trained research coordinators to further assess acceptability. This anonymous feedback allowed researchers to adjust methodology and better inform the generalizability, transferability, and effectiveness of this telehealth version of P4P for families. Parents were notified that this information would be recorded. Demographic information was also collected. Additionally, standardized report forms providing quantitative data were also collected pre- and post-intervention. This paper focuses on the creation of the methodology for the telehealth P4P program and general response (acceptability, feasibility) through qualitative feedback and therefore, it is not considered a mixed-methods paper as all quantitative data will be presented once data collection is complete.

### Data analysis

Demographic data were collected, tabulated, and presented descriptively. No differences were found between those who completed the course and those who did not. Thus, only data for intervention completers are indicated here. Attendance and homework completion were noted at each session and were deemed as adequate based on a cut-off determined by prior research. Questionnaire responses were evaluated as a ratio of the whole. Additional qualitative data, collected as described above, were analyzed using an inductive approach (55). This approach involved researchers amassing and tabulating all qualitative data, then identifying themes that emerged, and finally going back to the original responses and assessing goodness of fit and altering overarching themes as appropriate. Using an iterative, inductive strategy has been deemed effective and informative in qualitative analyses (56). Employing this approach allowed researchers to adopt a data-driven approach with reduced bias, where themes emerged from parents’ experiences rather than a top-down deductive approach based on previously held assumptions where researchers would have pre-existing ideas and constructs they aimed to capture. Further, using this qualitative, iterative methodology for continued analysis of feedback as more cohorts are completed to see if there are similar patterns or shifts in the experiences of parents and can further inform the dynamic themes that emerge.

## Results

### Data analysis

#### Acceptability and feasibility

Thirty families out of 36 completed the adapted telehealth program over three telehealth P4P groups. Families discontinued due to time constraints (e.g., new jobs, changes in schedules) and family medical concerns. This represents an 8.33% attrition rate, which is considered low for a group-based intervention and suggests high acceptability and feasibility of the program compared to other PEERS^®^ intervention papers. Average attendance was 14.6/16 sessions (range = 10–16 sessions attended), with 93.33% (28/30 families) completing 13 or more sessions and 83.33% (25/30 families) completing 14 or more sessions, which is considered adequate attendance for social skill gains, based on previous PEERS^®^ studies (13).

Homework compliance for targeted assignments (e.g., enrolling children in playgroups, organizing and facilitating playdates, recording video demonstrations of parent coached play using the *4 P’s*) was very high (with almost 100% of families completing satisfactory homework compliance each week). As noted above, though homework assignments changed for each weekly skill and when playgroups and playdates were introduced, individual assignments would often include attendance, practicing the skill of the week (e.g., asking a friend to play), submitting a video demonstrating the skill of the week, having a playdate, and enrolling in a playgroup. This further demonstrated strong feasibility and was on par, if not greater, than prior in-person P4P homework completion. Satisfactory homework compliance was considered completion of video sent in for first 5 weeks, and completing two out of three assignments for the subsequent weeks once there were additional assignments (e.g., having a playdate, enrolling in playgroup).

To further assess program acceptability, participants were asked their impressions in the anonymous Parent Satisfaction Questionnaire of the length of sessions (1.5 h weekly) and the program (16 weeks). Regarding length of each session, 18/28 parents (64.3%) said the length was just right, 8/28 (28.6%) said sessions were too long, and 2/28 (7.1%) said sessions were too short. Regarding P4P total program length, 19/28 parents (67.9%) said the length was just right and 9/28 (32.1%) said the program was too long. The latter finding might call into greater question the acceptability and tolerability of the intervention, though all participants who indicated the program length was too long (e.g., nine parents) completed the full program.

#### Satisfaction

Twenty-eight parents completed the post-intervention Parent Satisfaction Questionnaire, which assessed acceptability and satisfaction with the P4P telehealth parent-mediated format, and parent confidence. All questions were rated on a 1–5 Likert scale for the targeted constructs, which included confidence, helpfulness, satisfaction, and change in child social behavior (see Supplementary material for full Satisfaction Questionnaire). The majority of parents (92.9%, 26/28) were quite to very satisfied with the group (top >20% response options). All 28 parents found the components of the P4P telehealth groups (e.g., homework review, didactic lesson, parent discussion) to be somewhat to very helpful (100%, top >20% response options).

**FIGURE 2:**
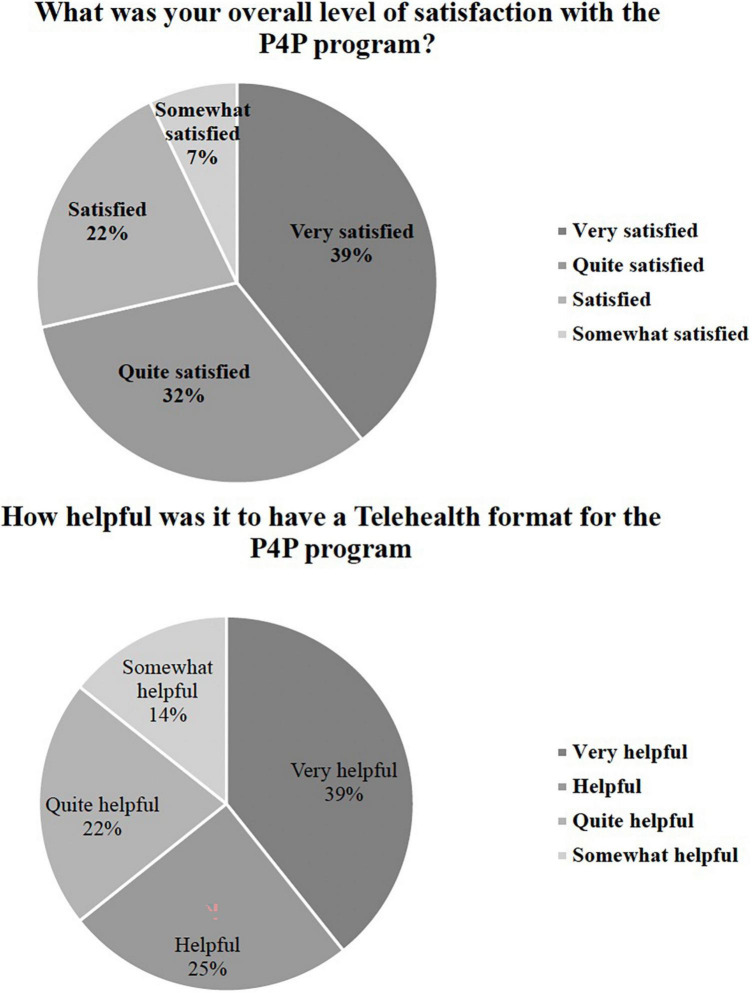
Satisfaction with P4P and telehealth adaptation. Only answers depicted in graphs/legends are those that had responses (i.e., options with 0 options are not presented), for all answer options, see Supplementary material below.

**FIGURE 3:**
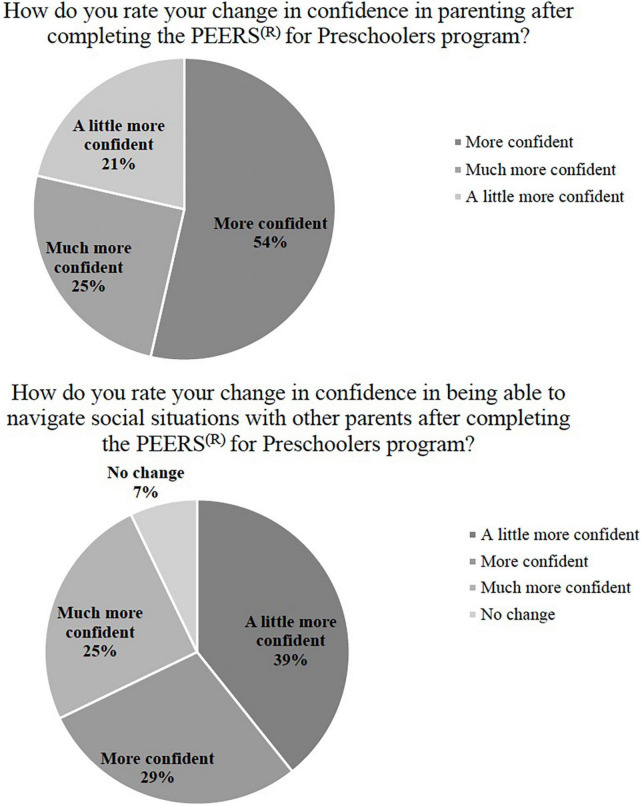
Parental confidence in social coaching child and with other parents. Only answers depicted in graphs/legends are those that had responses (i.e., options with 0 options are not presented), for all answer options, see Supplementary material below.

**FIGURE 4:**
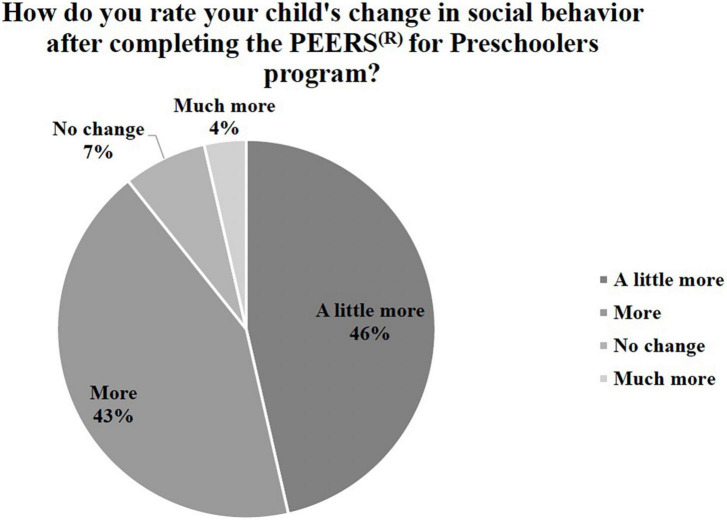
Child social skill change.

Furthermore, 100% of parents reported improvement in their confidence to coach their child in social skills and 92.9% reported more confidence in navigating social situations with other parents. Overall, 92.9% of parents reported a positive change in their child’s social skills *via* survey (Tables 3–5 and Figures 2–4).

**TABLE 3:** Satisfaction questions: Number and percent endorsed.

	Not satisfied		Somewhat satisfied		Satisfied		Quite satisfied		Very satisfied	
	Number parents endorsed	Percent parents endorsed	Number parents endorsed	Percent parents endorsed	Number parents endorsed	Percent parents endorsed	Number parents endorsed	Percent parents endorsed	Number parents endorsed	Percent parents endorsed
What was your overall level of satisfaction with the P4P program?	0	0%	2	7%	6	22%	9	32%	11	39%

	**Not helpful**		**Somewhat helpful**		**Helpful**		**Quite helpful**		**Very helpful**	
	**Number parents endorsed**	**Percent parents endorsed**	**Number parents endorsed**	**Percent parents endorsed**	**Number parents endorsed**	**Percent parents endorsed**	**Number parents endorsed**	**Percent parents endorsed**	**Number parents endorsed**	**Percent parents endorsed**

How helpful was telehealth PEERS^®^ for preschoolers program?	0	0%	4	14%	7	25%	6	22%	11	39%

**TABLE 4:** Satisfaction questions–confidence: Number and percent endorsed.

	Less confident		No change		A little more confident		More confident		Much more confident	
	Number parents endorsed	Percent parents endorsed	Number parents endorsed	Percent parents endorsed	Number parents endorsed	Percent parents endorsed	Number parents endorsed	Percent parents endorsed	Number parents endorsed	Percent parents endorsed
Change in confidence navigating social situations with other parents after completing P4P?	0	0%	2	7%	11	39%	8	29%	7	25%
Change in confidence in parenting after completing P4P program?	0	0%	0	0%	6	21%	15	54%	7	25%

**TABLE 5:** Satisfaction questions–child social skills: Number and percent endorsed.

	Less		No change		A little more		More		Much more	
	Number parents endorsed	Percent parents endorsed	Number parents endorsed	Percent parents endorsed	Number parents endorsed	Percent parents endorsed	Number parents endorsed	Percent parents endorsed	Number parents endorsed	Percent parents endorsed
Child’s change in social behavior on P4P skills after completing P4P program?	0	0%	2	7%	13	46%	12	43%	1	4%

#### Qualitative data analysis

Qualitative data were analyzed according to the methodology described above using an inductive, iterative approach (57, 58). Two coders reviewed the data and had over 90% interrater reliability in agreement regarding the codes. Based on this approach, four themes emerged: (1) improving child social skills (making and keeping friends), (2) parent confidence in social coaching, (3) ease of translation of P4P to telehealth, and (4) general response to P4P program. These themes are presented in Table 6 with supporting quotes and survey results included in Figures 2–4.

**TABLE 6:** Qualitative themes and selection of quotes.

Theme	Quotes
Improving child social skills (making and keeping friends)	• *“Now that we have these tools, it feels like we are finally having a successful playdate. So this was a huge success for my child and the family.”* • *“He did great following directions.”* • *“Before he would cry or get every emotional if we said no, but that doesn’t happen now. We notice that he sighs and says ‘Okay’ instead.”* • *“I feel like the process of this whole class has been an organic process and I don’t know exactly what he is improving on specifically, but I can tell he is improving.”*
Parent/caregiver confidence in social coaching	• *“It was very helpful to hear from other parents.”* • *“I really enjoyed when we broke up into groups and watched each other’s videos and were able to discuss and listen to other parents concerns and suggestions.”* • *“Again, it was very helpful to hear other parents’ experiences as a group and to have the professionals explain/suggest how to handle or act in that moment with your child and others.”* • *“Having a community of others with children who had similar issues was very helpful. It was good to learn from others and their experiences.”* • *“Keeping the conversations about playdates for the last weeks of the program was great. We learned a lot by listening to others and asking about ways to navigate difficult playdates. More solutions in that regard would helpful.”* • *“Discussions that involved other parents. Listening to their issues and concerns was very enlightening. You often feel alone when parenting a special needs child. Knowing that other parents are having similar issues is very reassuring.”* • *“Now that we have these tools, it feels like we are finally having successful playdates.”* • *“The training on how and when to provide feedback has really helped me coach my child.”* • *“The program is full of helpful information in navigating the world of parenting.”*
Ease of translation of telehealth	• *“The zoom format allowed me to attend.”* • *“This was a great experience. I am very thankful you had a remote session so folks like me who traveled could benefit from this resource. Thank you for all your do.”* • *“I loved the support and education provided by the UCLA PEERS program and really appreciate everybody’s efforts to help us learn this stuff even during the pandemic. The zoom sessions have been very convenient to attend. “* • *“I truly enjoyed the program and since it was online, via Zoom, I was able to attend this special program.”* • *“Connecting with other parents and seeing their videos. Some of the explicit conversation examples and ideas were helpful.”* • *“Although I liked the zoom session format and found it easy to attend, in person sessions would be very helpful for my child to get to have the social skills lessons taught and reinforced by the PEERS teachers as well as by me. Also, weekly in person social interactions with the other children in a controlled setting would probably be more helpful than me trying to set it up on my own which I have found challenging to do.”*
General response to the P4P program	• *“I really enjoyed watching the puppet shows with my child.”* • *“Reviewing the videos and getting coaching on my own parenting was so helpful!”* • *“The doctors’ feedback is what I’ll miss the most about these sessions!”* • *“The doctors’ feedback on the parents’ videos. They gave me an idea of what I was doing well at and helpful, easy to follow solutions/suggestions for problems I ran into. I found listening to the feedback on other parents’ videos helpful too.”* • *“The simplicity of the lessons and the printed one page format has been very helpful and easy to follow.”* • *“I loved the chat feature because it was nice to read other parents’ concerns as well as give and receive positive feedback from each other. Such a nice way to connect when we’re not able to meet in person or provide support for another parent who’s had an emotional moment when discussing their child.”* • *“You were all fantastic! My only regret is not having more time to dedicate to the program, but the materials will be something I will revisit for sure.”* • *“Reviewing the videos and getting coaching on my own parenting was so helpful!”* • *“I greatly appreciated the program and the facilitators. They were helpful, genuine and understanding. Thank you.”* • *“I’ve been praising my team members (at work) since PEERS and I think it makes for a happier team.”* • *“I loved learning about the importance of praise and really think PEERS has turned me into a much more effective praiser, with my kids but also life in general.”* • *“Overall a very beneficial program–I learned a lot from it and would highly recommend it to others. Thank you for this great service to the community!”* • *“Talking with the doctors and listening to other parents within the group setting was very helpful as well as getting specific feedback and coaching from the videos. The buzz words were life changing for our family!!”*

## Discussion

The current study is the first to our knowledge to extend an evidence-based social skills intervention (P4P) to a telehealth model for parents of young children with social difficulties. We provide a detailed methodological framework for the intervention (discussing the full implementation of this novel modality for P4P) and demonstrate feasibility and acceptability. Additionally, we demonstrate qualitative outcomes that highlight satisfaction with the telehealth P4P and improved parental confidence. Qualitative results suggest that the parent-mediated telehealth delivery of P4P is not only necessary, with constantly changing COVID-19 restrictions and longstanding other concerns regarding access to evidence-based interventions for young children with social difficulties, but viable. Overall, the present study highlights (1) the successful creation and implementation of a modified parent-mediated telehealth model for P4P, (2) acceptability and feasibility based on attendance, homework completion, and feedback, and (3) reported improved parental confidence and perceived child social skills after completion of the program. Based on these positive findings, researchers may be able to reach more families in need through the telehealth model of effectively teaching social skills.

Parents indicated the telehealth model was feasible and acceptable. This was shown through high retention and low attrition (30/36 families who started P4P completed P4P), high attendance maintained throughout the group (*M* = 14.6 sessions attended out of 16), and satisfactory homework compliance throughout P4P. Parents reported general acceptable length of each session (1.5 h), timing (weekday afternoon), and length of program (16 weeks). A few parents noted conflicts given timing and another requested longer sessions for greater discussion. In fact, some parents even created a means to communicate after the group through a group-chat.

Our findings indicate overall satisfaction with the creation and implementation of the telehealth parent-mediated P4P program. Almost all caregivers (26/28) were satisfied with the program, indicating high levels of fulfillment with this mode of intervention. This provides strong evidence for continuing to offer P4P using remote delivery. Additionally, all 28 caregivers who completed the survey found the distinct components (e.g., homework review, discussion) of the adapted P4P groups to be somewhat to very helpful (100%, >20% response options).

Specifically, parents noted they found the slides to be very helpful along with the parent-coaching videos, both distinct components of the telehealth adaptation and not provided during in-person groups. One factor that may have added to satisfaction is the notion that support for generalizability is built into this format by practicing with their children at home. Specifically, parents were not only able (and assigned) to practice skills in naturalistic settings (as they are during in-person groups), but were also provided support from the treatment team in refining their social coaching skills through the weekly viewing of video recordings of interactions. Therefore, practicing social coaching skills in natural settings (a standard treatment component of P4P homework assignments) with the added support of being able to debrief and refine coaching techniques is a unique aspect to P4P telehealth groups that may also have contributed to improved confidence. A number of parents suggested this feedback was the aspect of the program they found most effective.

Overall, parents rated an increase in their own confidence, both in social coaching their child and in social coaching in contexts with other parents. Specifically, 100% of parents reported improved confidence in their ability to coach their child in social skills and 93% indicated increased confidence in their ability to navigate social situations with other parents. This suggests that P4P may not only increase parent social coaching confidence in play settings, but may result in more global parent self-confidence (36). Parental self-efficacy (PSE), which predicts parent characteristics and behaviors, including competence and mental health (59), is an important concept for parents and caregivers of autistic children and those with social difficulties. Although most autistic children are diagnosed as preschoolers, symptoms often emerge earlier (1). Consequently, parents and caregivers may feel frustrated and even inept from employing ineffective techniques (34). Given the bidirectional relationship between child and parent in conjunction with the increased demand to involve parents in treatment, the benefits of P4P upon parents and parent-child relationships through increased PSE is promising, and further research is indicated (e.g., potential use of Parental Self-Efficacy in the Management of Asperger Syndrome, Parental Sense of Competence Scale, Parental Stress Index, etc.) (60).

Additionally, there was an observed increase in parent perceptions of child social skills following P4P treatment, even though the child did not receive direct intervention from the treatment team. Parent delivery of treatment through weekly viewing of puppet show role play videos and parent social coaching resulted in 93% of participants reporting a positive change in their child’s social skills. Although these findings are promising, additional research is needed to examine the specific mechanisms of change. Additionally, a number of parents indicated they would be interested in participating in an in-person version of the group as well to bolster skills learned. Qualitative themes suggest that this parent-focused telehealth approach does indeed improve child social skills (making and keeping friends), parent confidence in social coaching, and that the translation of P4P to telehealth is indeed feasible and effective. Further, this indicates the general positive response to the P4P program and material outlined in the paper and presented throughout the intervention.

Overall, these pilot findings are encouraging for the creation and implementation of the telehealth parent-mediated P4P program in providing young children foundational social skills to help navigate the social world (23). Initial feedback suggests that it is acceptable and feasible to administer P4P *via* telehealth using this model and there are additional benefits not seen in the in-person modality (e.g., through video feedback, more conversations with other parents). In the development of a social skills intervention, it is important to address perspectives of some in the autistic community. P4P targeted social skills that are not specific to autism and enrollment was not limited to a specific diagnostic group, as we recognize many individuals may seek out or want additional support learning social skills. With young children, some parents mentioned concern about “changing their children” or “not knowing how best to support their quirkiness or individuality.” Current research has suggested that some social skills interventions which seek to decrease autistic symptomatology may perpetuate a cycle of masking or camouflaging (e.g., fitting in) within the autistic community. Our goal in P4P and all PEERS^®^ programs is not to decrease neurodiversity or to change the autistic person, but rather to enhance the social interactions of socially motivated neurodivergent individuals.

We recognize the intrinsic motivation that some individuals have to interact with peers, while others do not, even at this young age. As one parent mentioned, “I want him to have the tools to interact socially if he wants to and I want to know how to best support him.” Additionally, encouraging parents to embrace their child’s individuality and interests is beneficial—we highlight that parents seek out playgroups based on their child’s interests so they can identify potential sources of friends who share common interests. We aim to teach skills that can alleviate social-communication differences that may become barriers to achieving social relationships within social contexts. Thus, children and parents are taught ecologically valid skills that may be beneficial in navigating the social world for any individual who struggles to make and keep friends. It is important to note that the decision to use these skills is a choice.

### Limitations

Although findings from this initial study are encouraging, it is not without limitations. As in most preliminary translation intervention research, group sizes were small, which limited data analysis. Future research on the larger P4P telehealth study will incorporate additional telehealth cohorts and allow for quantitative data analysis and comparison to in-person treatment groups as well. An RCT that employs a more rigorous study design would also allow for more definitive conclusions about P4P outcomes in assessing the delivery mechanism. The current methodology makes it difficult to rule out other variables as potentially impacting treatment findings. Moreover, the inclusion of more diverse samples [e.g., race, socioeconomic status (SES), gender] in future studies would promote the understanding of treatment impact across groups and the need for possible adaptations, as this sample was largely White, well-educated, with two-caregiver households. Thus, future research should include a more diverse sample to ensure the generalizability of this telehealth parent-mediated adaptation. Another limitation is the reliance on parent-report rather than observational measures (61). Parent involvement may bias post-treatment assessment of child social functioning (62), especially if parents believe they should respond a certain way to suggest positive results. Thus, observational data could add to the robustness of findings. Additionally, we only have parent report regarding diagnosis for this sample. Future studies will include a standardized testing measure regarding cognitive development, which will provide more information regarding the sample of children. We attempted to minimize bias by structuring the satisfaction questionnaire so answers would be anonymous though understand this does not alleviate the issue.

### Future directions

This pilot study presents a significant step in social skills intervention research for young children by documenting the creation and implementation of a remote delivery method during a global pandemic. We have demonstrated feasibility, acceptability, and initial positive satisfaction of this novel telehealth parent-mediated version of P4P, which is useful not only in mitigating the negative effects of COVID-19 access to services, but also the longstanding difficulties in accessing services for a variety of systemic reasons. Given the shift to include parents as active participants or even administrators in intervention (63), examining the efficacy of P4P is a critical next step. Additional standardized measures were also administered as part of ongoing data collection pre-and post-intervention, which will be examined in future research. We look forward to examining these quantitative measures to further explore outcomes from the P4P telehealth format, as well as exploring other environmental factors that would allow us to more acutely identify who this intervention would be most effective for and determine which factors would dictate positive response. Observational measures of child functioning as well as parent social coaching also need to be included in future work. As P4P continues to gain empirical support, adaptations for individuals with different needs (e.g., intellectual disability, non-verbal), and generalizability to other demographics, also need to be considered. Such considerations will enable researchers to increase dissemination efforts and reach a broader audience *via* virtual means and even hybrid P4P options.

## Conclusion

This study documents the creation and implementation of a modified, parent-mediated telehealth version of P4P, an evidenced based social skills intervention for young children. We demonstrate that this novel methodology can improve access to services and has documented acceptability, feasibility, and satisfaction in helping young children make and keep friends. Preliminary results support the use of this telehealth methodology of P4P, which also resulted in reported improved parent confidence and child social skills. Preliminary findings address a significant gap in the literature by demonstrating potential benefit of telehealth administered early social skills interventions to improve friendship skills for youth with social difficulties. Future research will enhance understanding of the efficacy of parent-mediated social skills intervention with larger and more diverse samples through RCTs using behavioral observational assessments of social functioning.

## Data availability statement

The raw data supporting the conclusions of this article will be made available by the authors, without undue reservation.

## Ethics statement

The studies involving human participants were reviewed and approved by the UCLA Institutional Review Board. The patients/participants provided their written informed consent to participate in this study.

## Author contributions

RF ran treatment groups, conceptualized the analyses, analyzed data, and wrote the manuscript. LG helped with running groups, conceptualizing the manuscript, and writing the manuscript. DB helped with running groups and writing the manuscript. EL created intervention and helped with reviewing the manuscript. All authors contributed to the article and approved the submitted version.

## Footnotes

1 Many self-advocates from the autism community (3) and current research has indicated both a preference for identify first language (e.g., autistic children) or language describing the individual (e.g., on the spectrum). Therefore, this language will be utilized throughout the present manuscript.

## References

[1] American Psychiatric Association [APA]. *Diagnostic and Statistical Manual of Mental Disorders: DSM-5.* 5th ed. Washington, DC: American Psychiatric Association (2013).

[2] Rao PA Beidel DC Murray MJ. Social skills interventions for children with asperger’s syndrome or high-functioning autism: a review and recommendations. *J Autism Dev Disord.* (2008) 38:353–61. 10.1007/s10803-007-0402-4 17641962

[3] Bury SM Jellett R Spoor JR Hedley D. “it defines who I am” or “it’s something I have”: what language do [Autistic] Australian adults [on the Autism Spectrum] prefer? *J Autism Dev Disord.* (2020). [Epub ahead of print]. 10.1007/s10803-020-04425-3 32112234

[4] Paul R. Promoting social communication in high functioning individuals with autistic spectrum disorders. *Child Adolesc Psychiatr Clin N Am.* (2003) 12:87–106. 10.1016/s1056-4993(02)00047-0 12512400

[5] Factor RS Ryan SM Farley JP Ollendick TH Scarpa A. Does the presence of anxiety and ADHD symptoms add to social impairment in children with autism spectrum disorder? *J Autism Dev Disord.* (2017) 47:1122–34. 10.1007/s10803-016-3025-9 28132125

[6] Bauminger N Kasari C. Loneliness and friendship in high-functioning children with autism. *Child Dev.* (2000) 71:447–56. 10.1111/1467-8624.00156 10834476

[7] Bauminger N Shulman C Agam G. Peer interaction and loneliness in high-functioning children with autism. *J Autism Dev Disord.* (2003) 33:489–507. 10.1023/A:1025827427901 14594329

[8] Kasari C Locke J Gulsrud A Rotheram-Fuller E. Social networks and friendships at school: comparing children with and without ASD. *J Autism Dev Disord.* (2011) 41:533–44. 10.1007/s10803-010-1076-x 20676748 PMC3076578

[9] Milton DE. On the ontological status of autism: the “double empathy problem. *Disabil Soc.* (2012) 27:883–7.

[10] Moody CT Laugeson EA. Social skills training in autism spectrum disorder across the lifespan. *Child Adolesc Psychiatr Clin.* (2020) 29:359–71. 10.1016/j.chc.2019.11.001 32169267

[11] Laugeson EA Frankel F Mogil C Dillon AR. Parent-assisted social skills training to improve friendships in teens with autism spectrum disorders. *J Autism Dev Disord.* (2009) 39:596–606. 10.1007/s10803-008-0664-5 19015968

[12] Laugeson EA Frankel F. *Social Skills for Teenagers With Developmental and Autism Spectrum Disorder: The PEERS Treatment Manual.* New York, NY: Routledge (2010). 10.2989/17280583.2013.802447 25860311

[13] Laugeson EA Frankel F Gantman A Dillon AR Mogil C. Evidence-based social skills training for adolescents with autism spectrum disorders: the UCLA PEERS program. *J Autism Dev.* (2012) 42:1025–36. 10.1007/s10803-011-1339-1 21858588

[14] Mandelberg J Laugeson EA Cunningham TD Ellingsen R Bates S Frankel F. Long-term treatment outcomes for parent-assisted social skills training for adolescents with autism spectrum disorders: the UCLA PEERS program. *J Ment Health Res Intellect Disabil.* (2014) 7:45–73. 10.1080/19315864.2012.730600

[15] DeRosier ME Swick DC Davis NO McMillen JS Matthews R. The efficacy of a social skills group intervention for improving social behaviors in children with high functioning autism spectrum disorders. *J Autism Dev Disord.* (2011) 41:1033–43. 10.1007/s10803-010-1128-2 21042870

[16] Tripathi I Estabillo JA Moody CT Laugeson EA. Long-term treatment outcomes of PEERS^®^ for preschoolers: a parent-mediated social skills training program for children with autism spectrum disorder. *J Autism Dev Disord.* (2022) 52:2610–26. 10.1007/s10803-021-05147-w 34302574 PMC9114088

[17] Wolstencroft J Robinson L Srinivasan R Kerry E Mandy W Skuse D. A systematic review of group social skills interventions, and meta-analysis of outcomes, for children with high functioning ASD. *J Autism Dev Disord.* (2018) 48:2293–307. 10.1007/s10803-018-3485-1 29423608 PMC5996019

[18] Maag JW. Social skills training for students with emotional and behavioral disorders: a review of reviews. *Behav Disord.* (2006) 32:4–17.

[19] Factor RS Rea HM Dahiya AV Albright J Ollendick TH Laugeson EA An initial pilot study examining child social skills, caregiver styles, and family functioning in the PEERS^®^ for Preschoolers program for young autistic children and their caregivers. *Res Dev Disabil.* (2022) 121:104152. 10.1016/j.ridd.2021.104152 34942441

[20] Factor RS Rea HM Laugeson EA Scarpa A. Examining feasibility and outcomes of the PEERS^®^ for preschoolers program. delivery. *J Autism Dev Disord.* (2022). [Epub ahead of print]. 10.1007/s10803-022-05502-5 35267147

[21] Park MN Moulton EE Laugeson EA. Parent-assisted social skills training for children with autism spectrum disorder: PEERS for preschoolers. *Focus Autism Other Dev Disabil.* (2022) 108835762211101. 10.1177/10883576221110158

[22] Kronberg J Tierney E Wallisch A Little LM. Early intervention service delivery via telehealth during COVID-19: a research-practice partnership. *Int J Telerehabil.* (2021) 13:e6363. 10.5195/ijt.2021.6363 34345340 PMC8287712

[23] Watkins L Kuhn M Ledbetter-Cho K Gevarter C O’Reilly M. Evidence-based social communication interventions for children with autism spectrum disorder. *Indian J Pediatr.* (2017) 84:68–75. 10.1007/s12098-015-1938-5 26581197

[24] Reichow B Volkmar FR. Social skills interventions for individuals with autism: evaluation for evidence-based practices within a best evidence synthesis framework. *J Autism Dev Disord.* (2010) 40:149–66. 10.1007/s10803-009-0842-0 19655240

[25] Gunning C Holloway J Fee B Breathnach Ó Bergin CM Greene I. A systematic review of generalization and maintenance outcomes of social skills intervention for preschool children with autism spectrum disorder. *Rev J Autism Dev Disord.* (2019) 6:172–99. 10.1007/s40489-019-00162-1

[26] Gates JA Kang E Lerner MD. Efficacy of group social skills interventions for youth with autism spectrum disorder: a systematic review and meta-analysis. *Clin Psychol Rev.* (2017) 52:164–81. 10.1016/j.cpr.2017.01.006 28130983 PMC5358101

[27] Kaat AJ Lecavalier L. Group-based social skills treatment: a methodological review. *Res Autism Spectr Disord.* (2014) 8:15–24. 10.1016/j.rasd.2013.10.007

[28] Boyd BA Conroy MA Asmus JM McKenney ELW. Direct observation of peer-related social interaction: outcomes for young children with autism spectrum disorders. *Exceptionality.* (2011) 19:94–108. 10.1080/09362835.2011.565724

[29] Gunning C Holloway J. Descriptive analysis of preschool social interactions. *J Behav Educ.* (2021) 1–27. 10.1007/s10864-020-09424-z

[30] Gengoux GW Schapp S Burton S Ardel CM Libove RA Baldi G Effects of a parent-implemented developmental reciprocity treatment program for children with autism spectrum disorder. *Autism.* (2019) 23:713–25. 10.1177/1362361318775538 29775078

[31] Rogers SJ Estes A Lord C Vismara L Winter J Fitzpatrick A Effects of a brief early start denver model (ESDM)–based parent intervention on toddlers at risk for autism spectrum disorders: a randomized controlled trial. *J Am Acad Child Adolesc Psychiatry.* (2012) 51:1052–65. 10.1016/j.jaac.2012.08.003 23021480 PMC3487718

[32] Bearss K Johnson C Handen B Smith T Scahill L. A pilot study of parent training in young children with autism spectrum disorders and disruptive behavior. *J Autism Dev Disord.* (2013) 43:829–40. 10.1007/s10803-012-1624-7 22941342

[33] Steiner AM Gengoux GW Klin A Chawarska K. Pivotal response treatment for infants at-risk for autism spectrum disorders: a pilot study. *J Autism Dev Disord.* (2013) 43:91–102. 10.1007/s10803-012-1542-8 22573001 PMC3571709

[34] Reichow B Steiner AM Volkmar F. Social skills groups for people aged 6 to 21 with autism spectrum disorders (ASD). *Cochrane Database Syst Rev.* (2012) CD008511. 10.1002/14651858.CD008511.pub2 22786515 PMC11975264

[35] Jurek L Occelli P Denis A Amestoy A Maffre T Dauchez T Efficacy of parent-mediated communication-focused treatment in toddlers with autism (PACT) delivered via videoconferencing: a randomised controlled trial study protocol. *BMJ Open.* (2021) 11:e044669. 10.1136/bmjopen-2020-044669 33827837 PMC8031029

[36] Karst JS Van Hecke AV. Parent and family impact of autism spectrum disorders: a review and proposed model for intervention evaluation. *Clin Child Fam Psychol Rev.* (2012) 15:247–77. 10.1007/s10567-012-0119-6 22869324

[37] Laugeson EA Gantman A Kapp SK Orenski K Ellingsen R. A randomized controlled trial to improve social skills in young adults with autism spectrum disorder: the UCLA PEERS§program. *J Autism Dev.* (2015) 45:3978–89. 10.1007/s10803-015-2504-8 26109247

[38] Garbarino J Dow-Burger K Ratner NB. Implementation of the program for the education and enrichment of relational skills (PEERS^®^) social skills intervention in a university-based communication sciences and disorders clinic. *Perspect ASHA Spec Interest Groups.* (2020) 5:637–45. 10.1044/2020_persp-20-00001

[39] McVey AJ Dolan BK Willar KS Pleiss S Karst JS Casnar CL A replication and extension of the PEERS^®^ for young adults social skills intervention: examining effects on social skills and social anxiety in young adults with autism spectrum disorder. *J Autism Dev Disord.* (2016) 46:3739–54. 10.1007/s10803-016-2911-5 27628940 PMC5310211

[40] Van Hecke AV Stevens S Carson AM Karst JS Dolan B Schohl K Measuring the plasticity of social approach: a randomized controlled trial of the effects of the PEERS intervention on EEG asymmetry in adolescents with autism spectrum disorders. *J Autism Dev Disord.* (2015) 45:316–35. 10.1007/s10803-013-1883-y 23812665

[41] Laugeson, Park, Bolton, Bolourian, Sanderson. A Randomized Controlled Trial of a Parent-Assisted Social Skills Treatment: The UCLA PEERS^®^ for Preschoolers Program. *2016. Poster abstract presented at the International Meeting for Autism Research.* Baltimore, MD (2016).

[42] Kamei A Harriott W. Social emotional learning in virtual settings: intervention strategies. *Int Electron J Elem Educ.* (2021) 13:365–71.

[43] Hanna N Lydon H Holloway J Barry L Walsh E. Apps to teach social skills to individuals with autism spectrum disorder: a review of the embedded behaviour change procedures. *Rev J Autism Dev Disord.* (2021) 1–17. 10.1007/s40489-021-00271-w

[44] Reed FD Hyman SR Hirst JM. Applications of technology to teach social skills to children with autism. *Res Autism Spectr Disord.* (2011) 5:1003–10.

[45] Shic F Goodwin M. Introduction to technologies in the daily lives of individuals with autism. *J Autism Dev Disord.* (2015) 45:3773–6. 10.1007/s10803-015-2640-1 26530715

[46] McCoy A Holloway J Healy O Rispoli M Neely L. A systematic review and evaluation of video modeling, role-play and computer-based instruction as social skills interventions for children and adolescents with high-functioning autism. *Rev J Autism Dev Disord.* (2016) 3:48–67. 10.1007/s40489-015-0065-6

[47] Estabillo JA Moody CT Poulhazan SJ Adery LH Denluck EM Laugeson EA. Efficacy of PEERS^®^ for adolescents via telehealth delivery. *J Autism Dev Disord.* (2022) 52:5232–42. 10.1007/s10803-022-05580-5 35624224 PMC9137447

[48] Drahota A Sadler R Hippensteel C Ingersoll B Bishop L. Service deserts and service oases: utilizing geographic information systems to evaluate service availability for individuals with autism spectrum disorder. *Autism.* (2020) 24:2008–20. 10.1177/1362361320931265 32564619 PMC7541434

[49] Ferguson J Craig EA Dounavi K. Telehealth as a model for providing behaviour analytic interventions to individuals with autism spectrum disorder: a systematic review. *J Autism Dev Disord.* (2019) 49:582–616. 10.1007/s10803-018-3724-5 30155578 PMC6373531

[50] Sutherland R Trembath D Roberts J. Telehealth and autism: a systematic search and review of the literature. *Int J Speech-Lang Pathol.* (2018) 20:324–36. 10.1080/17549507.2018.1465123 29709201

[51] Vismara LA McCormick C Young GS Nadhan A Monlux K. Preliminary findings of a telehealth approach to parent training in autism. *J Autism Dev Disord.* (2013) 43:2953–69. 10.1007/s10803-013-1841-8 23677382

[52] Hepburn SL Blakeley-Smith A Wolff B Reaven JA. Telehealth delivery of cognitive-behavioral intervention to youth with autism spectrum disorder and anxiety: a pilot study. *Autism.* (2016) 20:207–18. 10.1177/1362361315575164 25896267 PMC4615367

[53] Lindgren S Wacker D Suess A Schieltz K Pelzel K Kopelman T Telehealth and autism: treating challenging behavior at lower cost. *Pediatrics.* (2016) 137(Suppl. 2):S167–75. 10.1542/peds.2015-2851O 26908472 PMC4727312

[54] Kumm AJ Viljoen M de Vries PJ. The digital divide in technologies for autism: feasibility considerations for low- and middle-income countries. *J Autism Dev Disord.* (2022) 52:2300–13. 10.1007/s10803-021-05084-8 34121159 PMC8200284

[55] Miyake CJ Hong E Dixon DR Novack MN. Effectiveness of the Program for the Education and Enrichment of Relationship Skills (PEERS^§^) delivered via telehealth [Poster presentation]. *Association for Behavior Analysis International Annual Autism Conference* (2018).

[56] Wiltsey Stirman S Baumann AA Miller CJ. The FRAME: an expanded framework for reporting adaptations and modifications to evidence-based interventions. *Implement Sci.* (2019) 14:58. 10.1186/s13012-019-0898-y 31171014 PMC6554895

[57] Wertz FJ. *Five Ways of Doing Qualitative Analysis: Phenomenological Psychology, Grounded Theory, Discourse Analysis, Narrative Research, and Intuitive Inquiry.* New York, NY: Guilford Press (2011).

[58] Levitt HM Bamberg M Creswell JW Frost DM Josselson R Suárez-Orozco C. Journal article reporting standards for qualitative primary, qualitative meta-analytic, and mixed methods research in psychology: the APA Publications and Communications Board task force report. *Am Psychol.* (2018) 73:26–46. 10.1037/amp0000151 29345485

[59] Jones TL Prinz RJ. Potential roles of parental self-efficacy in parent and child adjustment: a review. *Clin Psychol Rev.* (2005) 25:341–63. 10.1016/j.cpr.2004.12.004 15792853

[60] Granger S des Rivières-Pigeon C Sabourin G Forget J. Mothers’ reports of their involvement in early intensive behavioral intervention. *Top Early Child Spec Educ.* (2012) 32:68–77. 10.1177/0271121410393285

[61] Whittingham K Sofronoff K Sheffield J Sanders MR. Stepping Stones Triple P: an RCT of a parenting program with parents of a child diagnosed with an autism spectrum disorder. *J Abnorm Child Psychol.* (2009) 37:469–80. 10.1007/s10802-008-9285-x 19023654

[62] White SW Keonig K Scahill L. Social skills development in children with autism spectrum disorders: a review of the intervention research. *J Autism Dev Disord.* (2007) 37:1858–68. 10.1007/s10803-006-0320-x 17195104

[63] Dixon L Lucksted A Stewart B Burland J Brown CH Postrado L Outcomes of the peer-taught 12-week family-to-family education program for severe mental illness. *Acta Psychiatr Scand.* (2004) 109:207–15. 10.1046/j.0001-690x.2003.00242.x 14984393

