# Supplementary material for: Remote PEERS® for preschoolers: A pilot parent-mediated social skills intervention for young children with social challenges over telehealth
Source: Front Psychiatry. 2022 Nov 29;13:1008485. doi: 10.3389/fpsyt.2022.1008485 (PMC9745198; doi:10.3389/fpsyt.2022.1008485)
Supplement: Supplementary file 1 [file Data_Sheet_1.pdf]

Supplementary Material

Satisfaction Questionnaire

Please answer each question as best as you can. Your answers will remain confidential.

1. What was your overall level of satisfaction with the program?

1	2	3	4	5
Not satisfied	Somewhat satisfied	Satisfied	Quite satisfied	Very satisfied

2. How helpful were the targeted social skills?

1	2	3	4	5
Not helpful	Somewhat helpful	Helpful	Quite helpful	Very helpful

3. How helpful were the homework assignments and review?

1	2	3	4	5
Not helpful	Somewhat helpful	Helpful	Quite helpful	Very helpful

4. How helpful was the zoom-session review of the videos you submitted with your child?

1	2	3	4	5
Not helpful	Somewhat helpful	Helpful	Quite helpful	Very helpful

5. Did you feel that the length of each session (90 minutes) was adequate?

1	2	3
Too short	Just right	Too long

6. Did you feel that the length of the program (16 weeks) was adequate?

1	2	3
Too short	Just right	Too long

7. How helpful was the telehealth format for the P4P program?

1	2	3	4	5
Not helpful	Somewhat helpful	Helpful	Quite helpful	Very helpful

8. How helpful were puppet show videos?

1	2	3	4	5
Not helpful	Somewhat helpful	Helpful	Quite helpful	Very helpful

9. How do you rate your change in confidence for parenting?

1	2	3	4	5
Less confident	No change	A little more confident	More confident	Much more confident

10. How do you rate your change in confidence in being able to navigate social situations with other parents?

1	2	3	4	5
Less confident	No change	A little more confident	More confident	Much more confident

11. How helpful was hearing from other parents in facilitated discussion?

1	2	3	4	5
Not helpful	Somewhat helpful	Helpful	Quite helpful	Very helpful

12. How do you rate change in social behavior on the P4P skills for your child?

1	2	3	4	5
Less	No change	A little more	More	Much more

13. What part of the program did you find most helpful? Please specify.

____

____

____

14. What suggestions do you have to improve the program?

____

____

____

Thank you for your feedback!
